# Superior eyelid lateral transorbital approach for resection of a Meckel’s cave schwannoma: how I do it

**DOI:** 10.1007/s00701-026-06866-8

**Published:** 2026-04-24

**Authors:** Mohamed Emara, Mohamed Samy Elhammady, Nora El Shammah, Florian Roser

**Affiliations:** 1 College of Medicine, University of Sharjah, Sharjah, UAE; 2 Department of Neurological Surgery, Neuroscience Institute, Cleveland Clinic Abu Dhabi, Abu Dhabi, United Arab Emirates; 3 Cleveland Clinic Lerner College of Medicine, Case Western Reserve University, Cleveland, OH USA; 4 Department of Ophthalmology, Integrated Surgical Institute, Cleveland Clinic Abu Dhabi, Abu Dhabi, United Arab Emirates

**Keywords:** Endoscopic, Schwannoma, Transorbital, Cavernous sinus, Meckel's cave

## Abstract

**Background:**

Trigeminal schwannomas are rare, benign, slow-growing nerve sheath tumors. Endoscopic Transorbital Approach (ETOA) utilization has gained traction in recent years.

**Method:**

The authors present a surgical video demonstrating the ETOA technique for resection of a Meckel’s cave schwannoma with cavernous sinus extension via a superior eyelid incision, with relevant surgical anatomy discussed.

**Conclusion:**

In carefully selected patients, the ETOA is a safe and effective minimally invasive surgical option that can address lesions involving the Meckel’s cave and cavernous sinus.

**Supplementary Information:**

The online version contains supplementary material available at 10.1007/s00701-026-06866-8.

## Case presentation

**Fig. 1:**
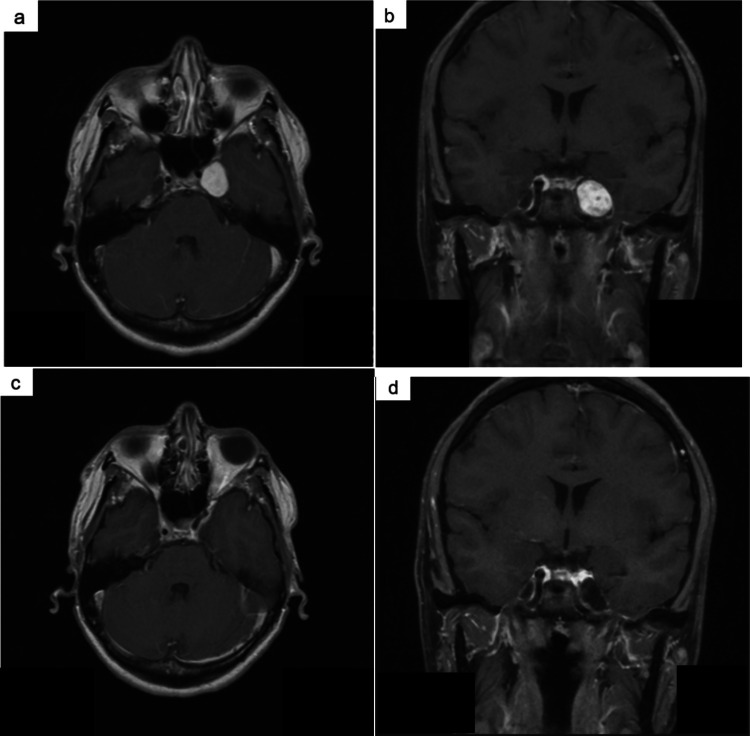
Preoperative axial (**a**) and coronal (**b**) T1 contrast brain MRI shows an enhancing left cavernous sinus lesion consistent with a trigeminal schwannoma. Postoperative axial (**c**) and coronal (**d**) T1 contrast brain MRI demonstrating complete resection of the lesion

The patient is a 42-year-old previously healthy female who was found to have an incidental left Meckel's cave lesion with extension to the cavernous sinus (Fig. 1a, b) during a workup for neck pain. On examination, the patient was neurologically intact. Management options including observation vs surgical resection vs radiosurgery were discussed with the patient. The patient ultimately decided to proceed with surgical resection through a transorbital route. Postoperative brain magnetic resonance imaging (MRI) demonstrated complete tumor resection (Fig. 1c, d). The patient developed a partial third nerve palsy following surgery, however at six-month follow-up she had normal extraocular movements without diplopia.

## Relevant surgical anatomy

Meckle’s cave is a dural CSF-filled space housing the trigeminal ganglion, serving as a conduit between the cavernous sinus and the prepontine cistern [4]. Tumors within Meckel’s cave may extend to the cavernous sinus [4]. Multiple transcranial and transsphenoidal approaches to the cavernous sinus exist, however ETOA provides a minimally invasive option to both Meckel's cave and the cavernous sinus [8].

As the lateral orbital wall is exposed, multiple anatomical variations can be encountered, especially with anastomotic vessels between the middle meningeal artery and the ophthalmic artery [3, 6]. A foramen through the greater wing of the sphenoid, known as foramen meningo-orbitale, Hyrtl’s canal, or cranio-orbital foramen, houses these vessels and is estimated to be present in approximately 42% of orbits [3, 6].

**Fig. 2:**
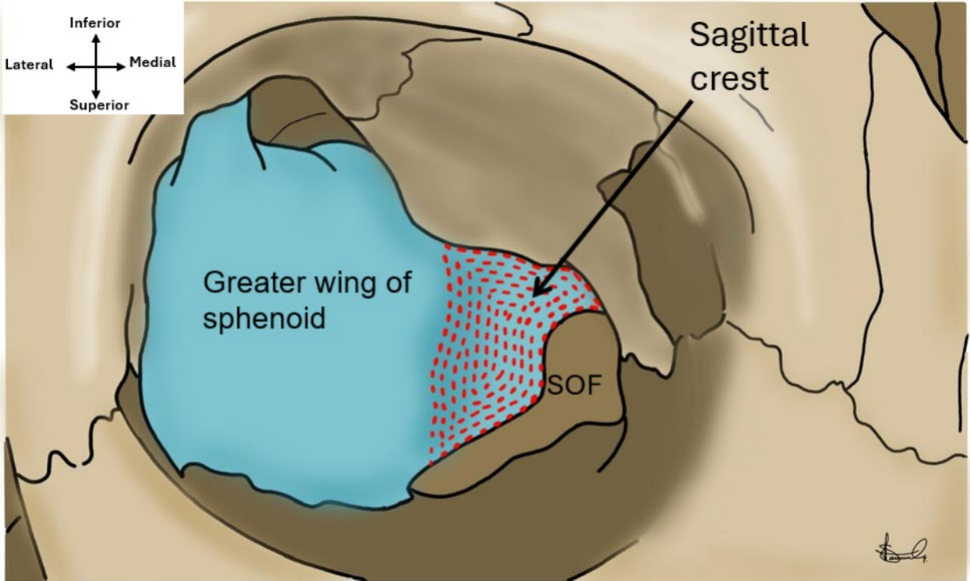
Approximation of the sagittal crest portion of the greater sphenoid wing; SOF, superior orbital fissure

The sagittal crest, (Fig. 2), is an artificially created triangular landmark as a result of lateral to medial drilling of the greater sphenoid wing [1]. The sagittal crest lies between the temporal dura and the periorbita [1]. It is composed of a base, anterior edge, and posterior edge [1]. The base denotes the exit of the V2 branch of the trigeminal nerve through foramen rotundum. The posterior edge of the sagittal crest delineated the interdural plane between the lateral wall of the cavernous sinus and the temporal dura [1]. The anterior edge is formed after the anterior surface of the greater wing of the sphenoid is removed [1].

## Description of the technique

### Positioning, equipment, and setup

The patient was positioned supine with her head slightly extended, rotated to the contralateral side, and secured in a 3-pin Mayfield head clamp. The primary surgeon stands at the head of the patient with the assistant surgeon and scrub technician on the left and right side of the patient, respectively.

### Incision and exposure of the lateral orbital wall

**Fig. 3:**
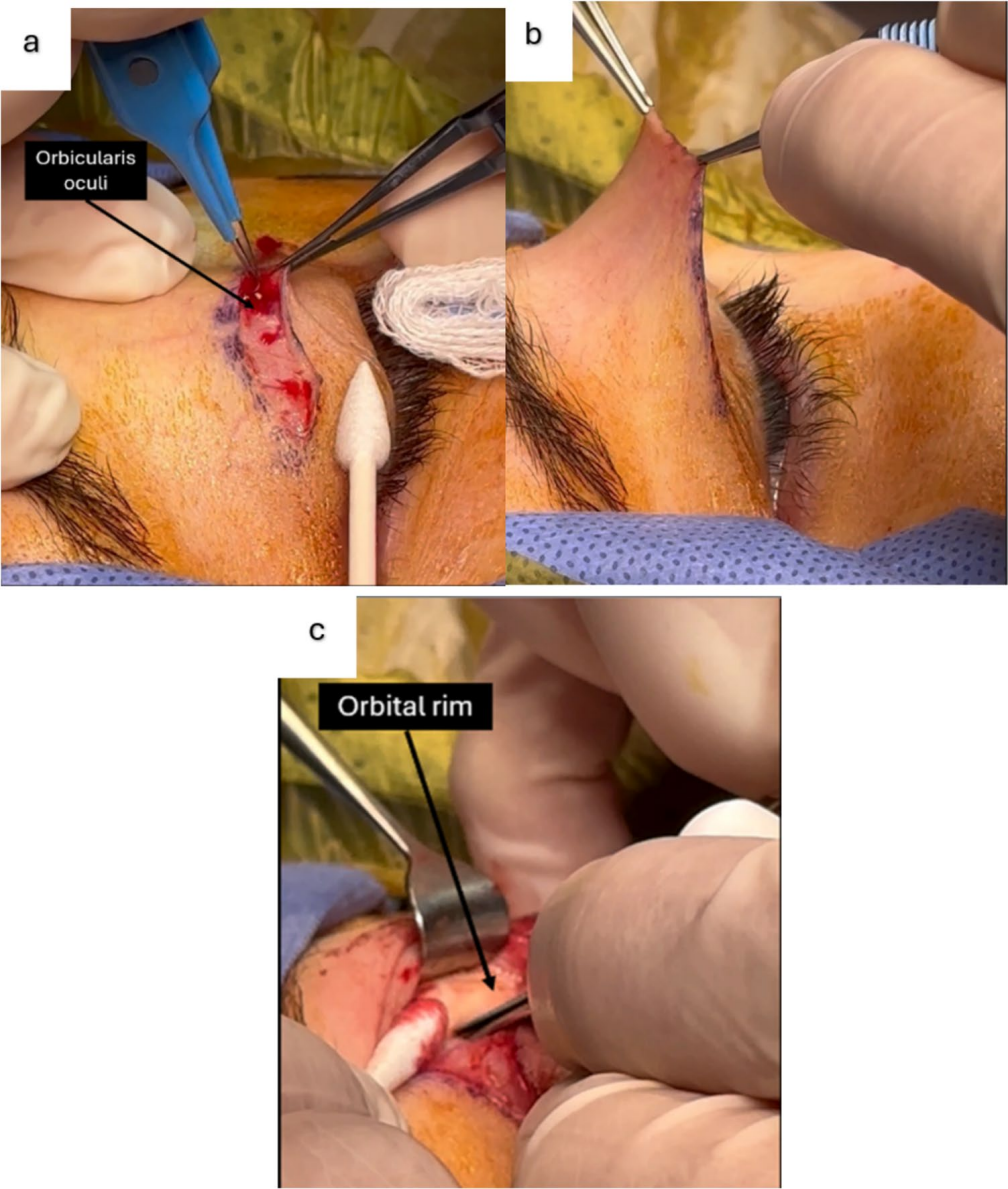
Initial exposure of the Orbicularis oculi. **a** Skin flap.** b** Exposure of the orbital rim. **c**

An upper eyelid incision with a skin flap was created with the help of an oculoplastic surgeon. The orbicularis oculi was dissected and the orbital rim exposed (Fig. 3).

The periorbita was dissected and then retracted medially with a rigid retractor. The endoscope was introduced. An orbital branch of the middle meningeal artery was identified, coagulated, and sharply sectioned to avoid avulsion. Further dissection of the periorbita posteriorly exposed the lateral orbital wall formed by the zygoma and greater sphenoid wing.

### Bone removal

The zygoma and greater sphenoid wing were subsequently drilled with a rough diamond burr until the temporalis muscle was exposed. Drilling was carried out further posteriorly, exposing the temporal dura. The meningo-orbital band (MOB), located at the lateral aspect of the superior orbital fissure, was exposed by drilling the greater and lesser wing of the sphenoid. The MOB was followed inferiorly, exposing the sagittal crest, which was then drilled away using a smaller drill bit.

### Cavernous sinus dissection, tumor resection, and reconstruction

The MOB was coagulated and divided. The temporal dura and lateral wall of the cavernous sinus were peeled away exposing the tumor capsule. Injectable haemostatic agents were used to control cavernous sinus bleeding. The tumor capsule was incised and internal debulking performed using ring curettes. After sufficient debulking was achieved the tumor capsule was dissected away from the trigeminal nerve rootlets. The tumor was then removed in a piecemeal fashion with pituitary rongeurs. Surgical cavity hemostasis was confirmed followed by reconstruction with an onlay dural substitute and fibrin glue.

### Indications

The ETOA can be utilized to address various pathologies involving the anterior, middle, and posterior cranial fossa [5, 8] such as tumours, abscesses, aneurysms, and fibrous dysplasia [2, 7, 8]. Furthermore, ETOA can be modified and combined with other surgical approaches to provide greater exposure and extended working space [8].

### Limitations

The ETOA provides a relatively narrow surgical corridor limited by the globe medially, which prevents aggressive retraction and may limit manoeuvrability. As discussed, multiple modifications to the approach exist to widen the surgical corridor, but still may not be sufficient for complex or extensive lesions. The use of ETOA for intracranial pathologies is relatively new and its advantages over traditional transcranial approaches have not been established.

### How to avoid complications

Visual complications can be minimized by avoiding excessive retraction of the orbital contents. For extradural pathologies, dural relaxation can be achieved with mannitol, hyperventilation, or use of a lumbar drain. Early identification, coagulation, and sectioning of the arteries running through Hyrtl’s canal avoids inadvertent avulsion and bleeding. Cavernous sinus bleeding can be controlled with injectable hemostatic agents.

### Specific information for the patient

In carefully selected patients, the ETOA is a minimally invasive, safe, and effective surgical procedure that avoids temporalis muscle wasting associated with traditional anterolateral transcranial approaches and nasal complications associated with transsphenoidal approaches. Potential risks associated with the ETOA include injury to orbital contents, cranial nerve palsies, and ICA injury.

## 10 key points summary

1. The ETOA is ideal for cavernous sinus and Meckel’s cave pathologies.
2. Intraoperative navigation is useful in identifying important anatomical landmarks.
3. The superior eyelid incision provides attractive cosmetic outcomes
4. Early identification, coagulation, and sectioning of the arteries running through Hyrtl’s canal avoids inadvertent avulsion and bleeding
5. Lateral orbital wall drilling exposes the temporalis muscle laterally and the temporal lobe dura posteriorly
6. The sagittal crest is an artificially created anatomical landmark formed by lateral to medial drilling of the greater sphenoid wing. The sagittal crest lies between the temporal dura and periorbita and allows identification of the V2 branch of the trigeminal nerve.
7. Coagulation and sectioning of the MOB facilitates dissection of the lateral wall of the cavernous sinus.
8. Central tumor debulking followed by capsule dissection helps reduce shear forces on delicate trigeminal rootlets with tumor manipulation.
9. In carefully selected patients, the ETOA is a minimally invasive, safe, and effective procedure, which avoids temporalis muscle wasting associated with transcranial approaches and nasal complications associated with transsphenoidal approaches.
10. A lumbar drain can be used in large tumors to achieve brain relaxation and facilitate dissection of the temporal lobe dura and lateral wall of the cavernous sinus.

## Supplementary Information

Below is the link to the electronic supplementary material.

ESM 1 Supplementary Material 1 (MP4 1.50 GB)

## Data Availability

Not applicable.

## Abbreviations

ETOA: Endoscopic Transorbital Approach
MRI: Magnetic resonance imaging
MOB: Meningo-orbital Band
CSF: Cerebrospinal Fluid
